# Whole-genome sequencing to understand the genetic architecture of common gene expression and biomarker phenotypes

**DOI:** 10.1093/hmg/ddu560

**Published:** 2014-11-06

**Authors:** Andrew R. Wood, Marcus A. Tuke, Mike Nalls, Dena Hernandez, J. Raphael Gibbs, Haoxiang Lin, Christopher S. Xu, Qibin Li, Juan Shen, Goo Jun, Marcio Almeida, Toshiko Tanaka, John R. B. Perry, Kyle Gaulton, Manny Rivas, Richard Pearson, Joanne E. Curran, Matthew P. Johnson, Harald H. H. Göring, Ravindranath Duggirala, John Blangero, Mark I. Mccarthy, Stefania Bandinelli, Anna Murray, Michael N. Weedon, Andrew Singleton, David Melzer, Luigi Ferrucci, Timothy M Frayling

**Affiliations:** 1 Genetics of Complex Traits, University of Exeter Medical School, Exeter, UK,; 2 Laboratory of Neurogenetics, National Institute of Aging, Bethesda, MD, USA,; 3 Department of Molecular Neuroscience and Reta Lila Laboratories, Institute of Neurology, UCL, London, UK,; 4 BGI-Shenzhen, Shenzhen 518083, China,; 5 Department of Biostatistics, University of Michigan, Ann Arbor, MI, USA,; 6 Genetics Department, Texas Biomedical Research Institute, San Antonio, TX, USA,; 7 Longitudinal Studies Section, Translational Gerontology Branch, Gerontology Research Center, National Institute on Aging, Baltimore, MD, USA,; 8 MRC Epidemiology Unit, University of Cambridge, Institute of Metabolic Science, Addenbrooke's Hospital, Cambridge CB2 0QQ, UK,; 9 Wellcome Trust Centre for Human Genetics, University of Oxford, Oxford, UK,; 10 Oxford Centre for Diabetes, Endocrinology and Metabolism, Churchill Hospital, Oxford, UK,; 11 Oxford National Institute for Health Research (NIHR) Biomedical Research Centre, Churchill Hospital, Oxford, UK,; 12 Tuscany Regional Health Agency, Florence, Italy,; 13 I.O.T. and Department of Medical and Surgical Critical Care, University of Florence, Florence, Italy,; 14 Geriatric Unit, Azienda Sanitaria di Firenze, Florence, Italy; 15 Institute of Biomedical and Clinical Sciences, University of Exeter Medical School, Barrack Road, Exeter, UK

## Abstract

Initial results from sequencing studies suggest that there are relatively few low-frequency (<5%) variants associated with large effects on common phenotypes. We performed low-pass whole-genome sequencing in 680 individuals from the InCHIANTI study to test two primary hypotheses: (i) that sequencing would detect single low-frequency–large effect variants that explained similar amounts of phenotypic variance as single common variants, and (ii) that some common variant associations could be explained by low-frequency variants. We tested two sets of disease-related common phenotypes for which we had statistical power to detect large numbers of common variant–common phenotype associations—11 132 *cis*-gene expression traits in 450 individuals and 93 circulating biomarkers in all 680 individuals. From a total of 11 657 229 high-quality variants of which 6 129 221 and 5 528 008 were common and low frequency (<5%), respectively, low frequency–large effect associations comprised 7% of detectable *cis*-gene expression traits [89 of 1314 *cis*-eQTLs at *P* < 1 × 10^−06^ (false discovery rate ∼5%)] and one of eight biomarker associations at *P* < 8 × 10^−10^. Very few (30 of 1232; 2%) common variant associations were fully explained by low-frequency variants. Our data show that whole-genome sequencing can identify low-frequency variants undetected by genotyping based approaches when sample sizes are sufficiently large to detect substantial numbers of common variant associations, and that common variant associations are rarely explained by single low-frequency variants of large effect.

## Introduction

Initial results from sequencing studies suggest that there are relatively few low-frequency (minor allele frequency <5%) variants associated with large effects on common human phenotypes (1–5). However, few of these sequencing experiments have used sample sizes similar to those required to identify most common variant–phenotype associations (1,4). Still fewer sequencing studies have examined the whole genome, instead most have focused on exomes (5) or targeted sets of genes (1,6) or have focused on population genetics rather than phenotype associations (7). Given that the proportion of phenotype variance explained is a function of allele frequency and effect size [approximated as *β*^2^ × (2*pq*)], limitations in sample size mean that many current sequencing studies are powered to detect only those single low-frequency variants that explain substantially more phenotypic variance than single common SNPs. In this study, we define ‘low frequency–large effect’ as a variant that has a minor allele frequency <5% but that has a sufficiently large per-allele effect on a phenotype that it explains a similar proportion of phenotypic variance as common variants detectable in the same sample size.

Current whole-genome and exome sequencing-based studies are aiming to answer several questions of relevance to common disease and quantitative phenotypes. First, how many low frequency and rare variant associations can we reasonably expect to identify and in what sample sizes? Second, are common variant–common phenotype associations driven by low frequency associations? Third, could we do just as well by imputing genotypes from the 1000 Genomes Project and other reference panels? These questions are important for a number of reasons. First, it is known that most human genetic variation is low frequency and rare but there is considerable debate as to how best to identify which of these variants are associated with common phenotypes. Studies include whole-exome and gene-targeted approaches (5,6) low-pass sequencing in unrelated individuals (4) and high-pass sequencing in families. Second, if low frequency and rare variants are responsible for many common variant–common phenotype associations it will likely implicate a different set of causal genes and regulatory elements for follow up. Finally, few studies have tested the power of imputation from reference panels to identify low-frequency association signals and this approach could be the most efficient way of studying lower frequency effects in large sample sizes, as recently shown by a deCODE study (8).

To help answer these questions we performed low-pass (median 7×) whole-genome sequencing in 680 individuals from the population based InCHIANTI study (Table 1; Supplementary Material, Fig. S1 and Table S1; Materials and Methods). We selected two sets of common phenotypes for which the InCHIANTI study provided sufficient statistical power to detect large numbers of common variant–common phenotype associations—11 132 whole-blood based *cis*-gene expression traits in 450 individuals, and 93 circulating biomarkers in 673 individuals. Previous microarray based GWAS of 1200 InCHIANTI individuals detected 1298 *cis*-eQTLs and 30 circulating biomarker associations (9,10). In addition to providing good power to detect common variant associations, these phenotypes are highly relevant to human disease. Multiple studies have shown that common variant disease associations are enriched for variants affecting gene expression in *cis* and *trans* (11–16) and our biomarkers included many of public health importance: vitamins A and D; cholesterol; magnesium, calcium and potassium ions; inflammatory markers and circulating proteins associated with metabolic disease (including leptin and adiponectin).

**Table 1.** Basic characteristics of the 680 InCHIANTI individuals selected for sequencing at baseline

Characteristic	Mean (range) or %
Age (years)	64.2 (23–90)
Sex (% male)	44.9%
Body mass index	27.2 (18.1–46.6)
Current smokers (% case)	21.3%
History of hypertension (% case)	33.8%
History of diabetes (% case)	8.7%
History of myocardial infarction (% case)	2.6%

We tested two main hypotheses. First, that whole-genome sequencing would detect single low-frequency genetic variants that individually explain a similar proportion of phenotypic variance as single common genetic variants. Our second main hypothesis was that some individual common variant–common phenotype associations would be explained by low frequency variants. As a secondary hypothesis that has recently been tested in other studies (7), we also tested whether or not low frequency variant–common phenotype associations would be better captured by low-pass sequencing than imputation from the 1000 Genomes Project.

## Results

**Figure 1.**
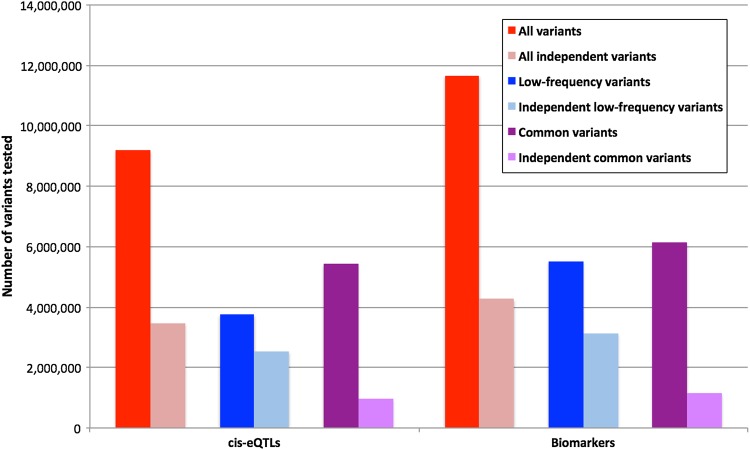
The total number of variants (low frequency and common) tested in the *cis*-eQTL and circulating biomarker analyses.

Analysis of our median 7-fold whole-genome sequencing data detected 11 657 229 high-quality variants (10 144 717 SNPs and 1 512 512 indels) (see Materials and Methods for definition of high quality) and had a minor allele count (MAC) ≥ 4. Of these variants 6 129 221, 5 528 008 and 2 917 071 had a minor allele frequency (MAF) ≥5% (common), MAF <5% (low frequency) and MAF <1% but MAC ≥ 4, respectively. We limited tests to those with a MAC ≥4 because we had limited power to detect associations with three or less alleles. A full break down of the numbers of variants tested for each of the analyses is shown in Table 2, Figure 1 and Supplementary Material, Tables S2 and 3.

**Table 2.** A breakdown of the number of variants with minor allele count ≥4 tested in the *cis*-eQTL and circulating biomarker analyses

Analysis	All Variants		MAF < 0.05		MAF ≥ 0.05	
	*N* variants tested	*N* estimated independent	*N* variants tested	*N* estimated independent	*N* variants tested	*N* estimated independent
*cis*-eQTLs	9 187 579	3 480 256	3 760 279	2 547 870	5 427 300	950 716
Biomarkers	11 657 229	4 272 000	5 528 008	3 127 500	6 129 221	1 167 000

Details of how we estimated the number of independent variants can be found in Materials and Methods.

A number of analyses provided strong evidence that our data were of high quality (Materials and Methods; Supplementary Material, Figs. S2–5 and Tables S4–7). We compared genotypes generated by low-pass sequencing with those identified from a separate targeted deep-sequencing (128×) experiment of 2 Mb of (non-contiguous) sequence from 83 overlapping individuals. This comparison provided an estimate that 99.4% of the variants identified by low-pass sequencing were true positives and a false negative (variants missed by low-pass sequencing but detected in deep-sequence data) rate of 11.9%. Equivalent figures for indels were in keeping with the increased difficulty of scoring these variants from low-pass sequencing data at 84 and 20.7%, respectively (Materials and Methods; Supplementary Material, Fig. S3 and Tables S4–7). Finally, a comparison between *non-reference* SNP genotypes generated by low-pass sequencing and GWAS chip data provided strong evidence that genotypes of common variants were accurately (mean genotype concordance of 99.7%) genotyped (Materials and Methods; Supplementary Material, Figs. S4 and 5).

All gene expression and biomarker phenotypes were inverse-normalized. A list of the 93 circulating biomarkers can be found in the Supplementary Material, Table S8. To assess the number of independent variants and tests we were performing, we randomly selected a 2 Mb region from each of the 22 chromosomes and used LDselect (17) and an *r*^2^ cut off of 0.8 to define independent signals (a likely conservative cut-off) (Materials and Methods; and Supplementary Material, Table S9). We conditioned all single common variant–phenotype associations on the most strongly associated single low-frequency variants within the locus (1 Mb either side), and vice versa conditioned all low frequency–phenotype associations on more strongly associated common variants in the same region (Materials and Methods). We used several approaches to test the robustness of our associations including testing *cis-*eQTL associations in a replication study—gene expression and high-pass (60×) whole-genome sequence data from 643 individuals from the San Antonio Family Heart Study (SAFHS) (Materials and Methods)—and validation of a subset of 11 low frequency variants with bespoke genotyping (Materials and Methods).

**Figure 2.**
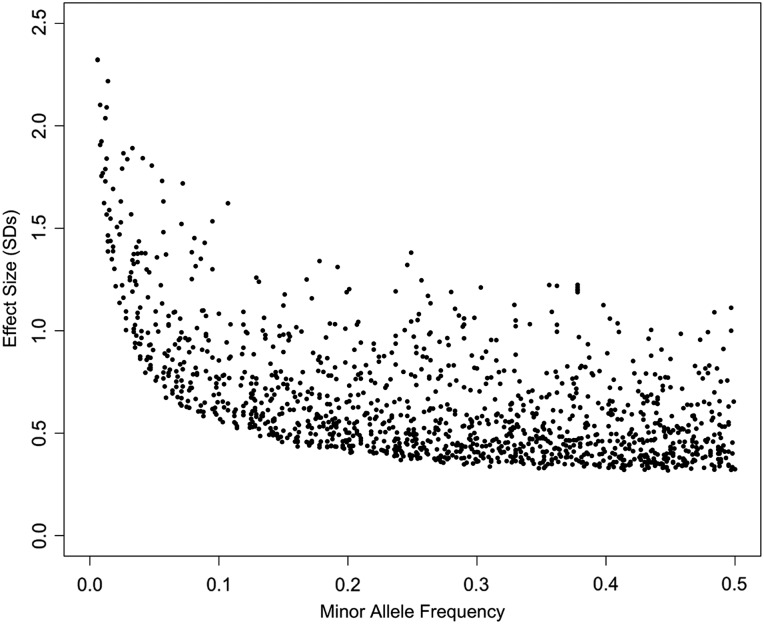
The distribution of effect sizes of index *cis*-eQTL variants by MAF.

We identified 1314 *cis*-eQTLs at *P* < 1 × 10^−06^ and 8 biomarker associations at *P* < 8 × 10^−10^, and for *cis*-eQTLs observed a continuous distribution between lower frequency variants of larger effect and higher frequency variants of smaller effect (Fig. 2 and Supplementary Material, Tables S10 and 11). Of the 6 129 221 common (>5%) and 5 528 008 low-frequency variants tested we identified 0.02 and 0.002%, respectively, as *cis*-eQTLs at *P* < 1 × 10^−06^. Low frequency–large effect associations comprised 7% of detectable *cis*-gene expression traits (89 of 1314) and one of eight biomarker associations. The average effect size of low frequency index variants was 1.36 (range 0.80–2.39) standard deviations and the average effect size of common index variants was 0.61 (range 0.32–1.73) standard deviations (Supplementary Material, Tables S10 and S11). These differences in per-allele effect size were expected given that lower frequency variants need to have larger per-allele effects to be detected (see Table 3 for power calculations).

**Table 3.** Statistical power to detect variants associated with gene expression in 450 individuals at *P* = 1 × 10^−6^ as a function of phenotypic variance explained and standard deviation (SD) effect size

MAF	Variance	Power	Effect (SD)	Variance	Power	Effect (SD)	Variance	Power	Effect (SD)
0.01	0.05	0.47	1.59	0.06	0.65	1.74	0.07	0.79	1.88
0.02	0.05	0.47	1.13	0.06	0.65	1.24	0.07	0.79	1.34
0.03	0.05	0.47	0.93	0.06	0.65	1.02	0.07	0.79	1.10
0.04	0.05	0.47	0.81	0.06	0.65	0.88	0.07	0.79	0.95
0.05	0.05	0.47	0.73	0.06	0.65	0.79	0.07	0.79	0.86
0.1	0.05	0.47	0.53	0.06	0.65	0.58	0.07	0.79	0.62
0.2	0.05	0.47	0.40	0.06	0.65	0.43	0.07	0.79	0.47

0.01	0.08	0.89	2.01	0.09	0.95	2.13	0.10	0.98	2.25
0.02	0.08	0.89	1.43	0.09	0.95	1.52	0.10	0.98	1.60
0.03	0.08	0.89	1.17	0.09	0.95	1.24	0.10	0.98	1.31
0.04	0.08	0.89	1.02	0.09	0.95	1.08	0.10	0.98	1.14
0.05	0.08	0.89	0.92	0.09	0.95	0.97	0.10	0.98	1.03
0.1	0.08	0.89	0.67	0.09	0.95	0.71	0.10	0.98	0.75
0.2	0.08	0.89	0.50	0.09	0.95	0.53	0.10	0.98	0.56

Our low-pass sequencing approach meant that we were able to accurately capture and analyze a similar number of low-frequency variants (5 528 008, including 2 917 071 at allele frequency <1%) as common variants (6 129 221). However, proportional to the number of variants analyzed, we detected far fewer low-frequency variant associations than common variant associations despite the same statistical power to detect individual variants explaining the same proportion of phenotypic variance. In Table 3 we show how statistical power remains fixed for variants explaining similar proportions of phenotypic variance, but how standard deviation effect sizes need to be higher for lower frequency variants. In total, we identified 89 low-frequency *cis*-eQTLs and 1 low frequency–circulating biomarker association compared with 1225 common *cis*-eQTLs and 7 common biomarker associations (Supplementary Material, Tables S10 and 11). Accounting for linkage disequilibrium between variants accentuated this difference—for *cis*-eQTLs the low-frequency variant associations represented 0.003% of an estimated 2 547 870 independent low-frequency variants (where independence was defined as *r*^2^ < 0.8), while the common variant associations represented 0.12% of an estimated 950 716 independent common variants (*r*^2^ < 0.8) (Table 2 and Fig. 1). These comparisons were not influenced by differences in quality of genotypes between low frequency and common variants because we only compared variants of high quality. Under an alternative genetic architecture, we could have expected to identify 1105 low-frequency variants associated with *cis-*gene expression (0.02% of 5 528 008 low-frequency variants tested—the same proportion of common variants associated with gene expression), given we had the same statistical power to detect single low frequency–large effect variants that explain a similar proportion of phenotypic variance as single common smaller effect variants. Instead our data are consistent with the argument that only a small proportion of single low-frequency variants will have large enough per-allele effects to explain a similar proportion of phenotype variance as single common variants. Our data do not rule out the possibility that many 1000s of low-frequency variants of moderate and small effect could collectively account for more phenotype variance than common variants collectively.

Analyses of gene expression phenotypes in a second dataset suggested that the associations observed were robust—of 233 *cis*-eQTLs associations where the same gene was probed with the same expression probe sequence in a second study of similar size (*N* = 643 related individuals, Materials and Methods), we detected 166 associated at a Bonferroni corrected *P*-value of <0.0002 and 222 of 236 were directionally consistent. Of these 12 of 17 testable low-frequency associations reached *P* < 0.0002 and all 17 were directionally consistent. For example, low-frequency variants in or near the genes *ACAD9*, *HDHD3*, *SOS1, UTS2*, *RTN1* and *RBPMS2* influenced the expression of those genes with per-allele effects of >1 standard deviation in the replication data, where winner's curse would not have appreciably influenced the effect size. No data were available to replicate the single low-frequency variant associated with a biomarker. This biomarker was lactate dehydrogenase and we could not identify any studies with relevant measures (Supplementary Material, Table S12).

We next assessed the extent to which common variant associations were driven by low-frequency associations, and vice versa, by conditioning on the most strongly associated variants in the alternative allele frequency bin. All evidence of association was lost (*P* > 0.05) for 13 of 1232 common variant associations when conditioning on the strongest low frequency variant and 969 of 1232 remained associated with our statistical thresholds. We next repeated the analyses but conditioned all common variant associations on all independent (*r*^2^ < 0.2) low-frequency variants reaching *P* < 1 × 10^−04^ in the *cis* region of the expression probe or within 1 Mb of the common index variants for biomarker associations. All evidence of association was lost (*P* > 0.05) for 30 of the 661 (5%) common signals that had at least one low-frequency association at the same locus. These results strongly suggested that few of the common associations were driven by single or multiple low-frequency variants. For low-frequency variants, all evidence of association was lost (*P* > 0.05) for 11 of 90 associations and 47 of the 90 remained associated at our thresholds when adjusting for the strongest common variant in the region (46 of the 89 low frequency *cis*-eQTL signals and 1 of 1 low-frequency biomarker associations) (Supplementary Material, Tables S13 and 14).

The availability of 1000 Genomes reference sequence has improved the ability to accurately capture low-frequency variants and may mean that low-pass sequencing individual studies is an inefficient use of research funds. We therefore next assessed how well low-frequency variant associations would have been detected without any sequence data from the InCHIANTI study but by using data from the 1000 Genomes Project as a reference panel for imputation and the GWAS array (Illumina HumanHap550K) genotypes as a scaffold. We used an imputation reference panel comprised of 2184 haplotypes from 1092 individuals sequenced and phased by the 1000 Genomes Project. Of these, 379 individuals were of European descent and included 98 individuals from Tuscany (the same part of Italy as the InCHIANTI study)*.* Of the 90 low-frequency signals (89 *cis*-eQTLs, 1 biomarker), detected by sequencing, we did not detect 63% (57) based on the same statistical thresholds using 1000 Genomes imputation alone (all *cis*-eQTLs) (Supplementary Material, Tables S15 and 16). Ignoring statistical thresholds, 85% of all *cis*-eQTL and biomarker associations identified through sequence-based analysis were less strongly associated in the 1000 Genomes imputed dataset or had no proxy within 250 Kb of the index variant (Supplementary Material, Tables S15 and 16). However, 62 (69%) of the 90 low-frequency variant associations were detected at *P* < 0.0001, illustrating that imputation from the 1000 Genomes reference panel captures most of the low-frequency variants, just not as accurately.

## Discussion

Our study provides an early example of a whole-genome sequencing experiment designed to identify low-frequency variants associated with common human phenotypes. We could accept our first main hypothesis. We show that, when using the same sample size for detecting both common and low frequency variants, whole-genome sequencing has the ability to identify low frequency variants with larger effect sizes (and similar phenotypic variance explained) than those observed for common variants. However, our data suggest that these single-variant low-frequency large effect signals may represent a relatively small proportion (here 7%) of detectable associations. Our data are consistent with the majority of single low-frequency genetic variants explaining smaller proportions of phenotypic variance than single common genetic variants. This result was perhaps expected, but given our sequencing-based approach allowed us to test a similar number of high-quality low-frequency variants (5 528 008) as common variants (6 129 221) we could reasonably have expected to identify ∼1105 low-frequency *cis*-eQTLs under a different genetic architecture. If extrapolated to other common human phenotypes, our results would indicate that, for example, whole-genome sequenced (or perhaps extremely well imputed) sample sizes of 35 000 cases and equivalent controls will be needed to detect ∼5 single low-frequency variants associated with type 2 diabetes (7% of 65, ref. 18). These are obviously very cautious extrapolations because the genetic architecture of *cis-*gene expression and circulating biomarkers may be very different compared with other phenotypes. Nevertheless, there is strong evidence that changes to *cis-*gene expression is a common mechanism leading to common disease and quantitative phenotypes (11–16) and a recent whole-genome sequence and imputation-based study provides evidence that these estimates may be of the correct order of magnitude—an effective sample size of 13 500 type 2 diabetes cases detected five low frequency (MAF < 5%) large effect type 2 diabetes associations (8).

Our data enabled us to largely reject our second main hypothesis—that common variant–common phenotype associations are explained by individual or multiple low-frequency variants. Only 30 (2%) common variant associations were driven by low frequency (and by necessity larger effect) variants. In contrast, a larger fraction (12%) of low-frequency variants was entirely driven by common variant associations at the same locus. We also note that, as with most sequencing studies, low-frequency insertion/deletion variants were harder to call and we may have missed true associations caused by these types of variant. Of note, 13% (12) of the 90 low frequency–large effect and 11% (137) of the 1232 common variant association signals included an indel as the most strongly associated variant.

In keeping with other recent studies we were able to accept our secondary hypothesis. Our data clearly show that whole-genome sequencing is more effective than imputation from the current 1000 Genomes Project reference panel. Imputation of missing genotypes missed 63% of low frequency–large effect associations detected by whole-genome sequencing at *P* < 1 × 10^−6^, and 31% at *P* < 1 × 10^−4^. Nevertheless, larger reference panels will improve the ability of imputation to capture low-frequency variants and it is notable that 1000 Genomes imputation captured at least half the genotype information (*r*^2^ > 0.52) for 75% of the low frequency signals. As noted by other studies (7), these findings emphasize the need for larger reference panels from which to impute missing genotypes into extremely large GWAS datasets.

There were a number of limitations to our study. First, our conclusions are based largely on testing 1000s of *cis-*gene expression phenotypes rather than the whole genome for a small number of phenotypes. However, the costs of whole-genome sequencing have so far limited single study sample sizes to <3000 samples, and our approach had the advantage that we were well powered to detect many common variant–common phenotype associations. This advantage meant we were able to make a fair comparison between common and low-frequency variants. Our data may also be relevant to disease phenotypes because numerous studies have shown that many disease associations are enriched for *cis-*gene expression effects (11–16). A second limitation is that we did not assess more detailed phenotypes or genotype combinations. For example, a recent study has shown that low-frequency variants may be a frequent cause of allele and exon-specific changes to gene expression (19). Furthermore we could not test the role of most rare variants (<0.5%) in our study because our sample size limited analyses to those occurring at 0.3% frequency or more (minor allele count ≥4). However, our approach meant we were able to analyze 2.9 million accurately called variants with allele frequencies of <1%.

In conclusion, our approach provided an unbiased assessment of the relative contribution of low frequency and common genetic variation to common quantitative phenotypes of relevance to human disease. Our study shows that low-pass whole-genome sequencing can identify low frequency–large effect variants in common human phenotypes using sample sizes sufficiently large to provide statistical power to detect large numbers of common variant associations.

## Materials and Methods

### Samples

We selected 680 individuals from the InCHIANTI study (9,20); a study of aging from the Chianti region in Tuscany, Italy, for low-pass whole-genome sequencing (Table 1). Selection criteria included the availability of microarray genotype data and non-missingness of phenotypic data that included gene expression data and circulating biomarker.

### Whole-genome sequencing

Whole-genome sequencing was performed at the Beijing Genomics Institute (BGI), Shenzhen, China using Illumina HiSeq 2000 to obtain a minimum read depth of 6× and median of 7×. An average of 240 million paired-end 90 bp reads per sample were aligned to the 1000 Genomes implementation of the build 37 genome reference consortium human reference genome (21), using the burrows-wheeler aligner (BWA) version 1.5.9 (22) (Supplementary Material, Fig. S1 and Table S1).

### Sequence read processing

Using the sequence reads aligned at BGI through BWA, each genome was scanned for small insertions and deletions (indels) using the Genome Analysis Toolkit (GATK) version 1.6 indel re-aligner (23). This process detected both de novo and known indels from dbSNP version 135 (24). Regions containing indels were then realigned to the reference genome. Duplicated reads across the genome were detected using Picard version 1.59 (available from http://picard.sourceforge.net) and subsequently removed to avoid potential bias when genotyping. In addition, base quality scores in each aligned read were recalibrated using the GATK version 1.6 table recalibrator. Recalibration used read group, reported quality score, sequencing machine cycle and sequence context as covariates.

### Sequence variant identification

SNP and indel calling was performed across all 680 genomes using the GATK version 2.2 unified genotyper. False-positive variant calls were filtered using variant quality score recalibration (VQSR). VQSR developed a covarying estimate of the relationship between eight variant call annotations (read depth, mapping quality, quality of read depth, haplotype score, inbreeding coefficient, mapping quality bias, strand bias and read position bias) and the probability that the call is a true genetic variant. The truth model was determined adaptively based on HapMap 3.3 sites and polymorphic sites from the 1000 genomes Omni 2.5 M SNP chip array (25,26).

### Quality control of variant capture and sequence-based genotype calls

As a quality control check we first used GATK's variant annotator (version 2.2) to determine the overlap of discovered variants cataloged in HapMap 3.3 (26), 1000 Genomes Omni 2.5 and the 1000 Genomes phase 1 indel dataset (25) (Supplementary Material, Fig. S2).

### Imputation of sequence data to recover and refine genotype sites

Haplotype phasing was performed using Beagle version 3.3 (27), and missing data were imputed internally using the filtered and present genotypes only. For SNPs we observed an overall Ti/Tv of 2.19. A summary of the variants captured can be found in the Supplementary Material, Tables S2 and S3.

### Variant and genotype comparison with 2 Mb of high depth sequence (median >30×)

We compared variants captured though our low-pass sequencing experiment with regions known to associated with Parkinson's disease sequenced at high depth (median >30×) in 96 InCHIANTI subjects (total of ∼2 Mb). SNP and indel calling was performed across the 96 samples using the GATK's unified genotyper (version 2.2). SNPs were filtered using VQSR and indels were hard filtered using GATK version 2.2 variant filtration. Of the 96 subjects, 83 subjects overlapped formed a subset of the 680 whole-genome sequenced subjects.

To assess the quality of the variants captured and the genotypes called in low-pass sequencing we created a high quality set of variants and genotypes called in the high depth 2 Mb of sequence data. We filtered by (1) masking out polymorphic regions in chromosome 6 and 17 in the 2 Mb regions in both datasets; (2) removing sites containing a genotype called at <20× coverage in the high-depth sequence dataset from both datasets; (3) removed all non-biallelic sites from the respective dataset.

The degree of overlap was then calculated each way for both of the filtered 2 Mb datasets using the GATK version 2.2 variant annotator and the genotype concordance matrices were calculated in overlapping sites using VCFtools version 0.1.9 (28) (Supplementary Material, Fig. S3 and Tables S4–7).

### Quality control of genotypes derived from sequence-based imputation

As an additional quality control check of internally imputation genotypes we performed genotype concordance checks with the Illumina HumanHap550 GWAS chip. Of the 680 subjects, 7 were selected for exclusion as the fraction of concordant genotypes for each subject was consistent with a sample swap (52% concordance in each instance). For the remaining 673 subjects we observed good concordance with the genotyping array (>98% concordance). For all 673 samples genotyping calls increased after internal imputation performed by Beagle (Supplementary Material, Figs. S4 and 5).

### 1000 Genomes imputation

To compare whole-genome sequencing to imputation from the 1000 genomes reference panel we used haplotypes from the 1000 Genomes Phase I integrated (version 3) release with singletons removed (30 061 896 variants; 28 681 763 SNPs and 1 380 133 indels). Genotype data captured on the Illumina HumanHap550 chip were phased using MACH 1.0.16 (29,30). Subsequent imputation was performed using Minimac (version 2012.10.9) (31). We used a multi-ethnic haplotype reference panel that included 1092 individuals including 379 Europeans (including 98 Tuscans), 181 Americans, 246 Africans and 289 Asians, in an attempt to capture variants that may be rare in Europeans but more common on haplotypes from different ethnic backgrounds.

### Variants included in association analyses

For all association analyses we filtered on biallelic variants with a minor allele count ≥4 and an *r*^2^ imputation quality >0.7. To ensure comparable imputation metrics between the Beagle- and MaCH/Minimac-derived dosages we recalibrated the Beagle imputation metric to MaCH's *r*^2^ (30). As described above, Beagle was used to refine and recover genotypes for the variant sites identified by the low-pass sequencing data.

### *cis*-eQTL association analysis

Whole-genome expression profiles of the InCHIANTI subjects were derived from whole blood and captured using the Illumina HT12-v3 BeadChip as previously described (32). We excluded probes that harbored non-singleton variants within the 50 bp probe region captured by our sequencing efforts or the Exome Sequencing Project (33). This resulted in 11 132 probes for association testing. We performed kinship analysis using KING (34) and removed first-degree relatives from the analysis that resulted in 450 remaining individuals. We inverse-normalized the intensity values for the filtered probes and individuals prior to generating residuals that adjusted for age, sex, amplification batch and hybridization batch to increase the likelihood of the error around the model being normally distributed. Finally, we inverse-normalized the residual values prior to performing the association analyses. We performed association testing in *cis* having defined a *cis* region as ±1 Mb the probe transcription start site. Dosages output by BEAGLE were formatted for MACH2QTL (29,30) and variants in *cis* tested against the normalized intensity values of the respective probe.

### Circulating biomarker association analyses

A full list of the 93 circulating biomarkers is provided in the Supplementary Material, Table S8. For the 93 circulating biomarkers we similarly performed a double inverse-normalization for each trait but inversed normalized the raw data values, and adjusted for age and sex only when generating the residuals. We tested the entire genome for associations against each of the circulating biomarkers using all 673 chip-concordant subjects. We used a mixed-linear model as implemented in EMMAX (35) to account for relatedness instead of removing subjects from the analysis.

### Estimating numbers of independent variants using 2 Mb windows

We used LDSelect version 1.0 (17) across 22 2 Mb windows (one per autosomal chromosome) to estimate the average number of independent variants (MAC ≥ 4) we would expect to observe, defining variants as independent if their pair-wise *r*^2^ cut-off <0.8. We estimated an average total of 2848 independent variants within a 2 Mb window (Supplementary Material, Table S9). In addition, we observed an average of 2085 and 778 low-frequency and common independent variants, respectively, within a 2 Mb window. Only those variants with imputation quality >0.7 were included in this analysis. Using this information, we estimated the number of independent variants tested in the association analyses. As 2 Mb represents ∼1/1500 of the genome we extrapolated estimates for the number of independent variants for the circulating biomarkers (all and split by minor allele frequency bin) multiplying by 1500.

### Calculating statistical thresholds for association analyses

For *cis*-eQTLs analyses there were a total of 9 187 579 analyzable variants (MAC ≥ 4 and imputation *r*^2^ > 0.7) that fell within 11 132 2 Mb windows around each of the gene expression probes. Given the estimated number of independent variants within a 2 Mb region was 2848 we calculated 2848 × 11 122 gene expression phenotypes = 31 675 456 independent tests. A *P*-value of 1.6 × 10^−9^ provides a Bonferroni corrected *P*-value of 0.05 and a *P*-value of ∼1 × 10^−06^ provides a false-discovery rate of ∼5% given the number of *cis*-eQTLs we identified at that threshold (1314).

For the 93 circulating biomarkers we first estimated the number of independent variants across the whole genome by multiplying the number of independent (*r*^2^ < 0.8) variants in a 2 Mb window, 2848, by the approximate number of 2 Mb windows, 1500 = 4 272 000. We multiplied this number by number of circulating biomarkers we were testing to give a total of 397 296 000 independent tests. A *P*-value of 8 × 10^−10^ provides a Bonferroni corrected *P*-value of 0.05.

### Conditional analysis using variants in opposing minor allele frequency bins

For associations that reached our statistical thresholds, we conditioned on the dosage of the most significant variant from the opposing MAF bin (MAF <5 versus MAF ≥ 5%). These variants were limited to those either within the 2 Mb *cis* region of the specific expression trait or within 1 Mb of the index variant representing a circulating biomarker association. To ensure that a lack of change in significance of the index variant was not driven by the best variant from the opposite minor allele frequency bin belonging to a secondary signal (creating the potential to miss a partially tagging variant that may not have been the most significant in the opposing bin), we performed full conditional analysis on all traits and conditioned the original index variant identified on the best variant from the opposing allele frequency bin from all additional signals that were observed.

To test further whether or not low-frequency variants could explain common signals, we conditioned common signals on all independent low frequency variants (*r*^2^ < 0.2) with *P* < 1 × 10^−4^ within the region. Association-based variant clumping was performed using PLINK (36) to identify the variants required for this conditional analysis. Of the 1232 common signals, 661 had ≥1 low frequency variant in the region meeting these criteria. Three hundred and thirty common signals had two or more low-frequency variants that we conditioned on.

### Replication of *cis*-eQTLs in the SAFHS

Whole-genome expression profiling was performed using Illumina Sentrix Human Whole Genome (WG-6) Series I as previously described (37) and called genotypes and were provided by the T2D-GENES Consortium. Genotypes were derived either directly from high-pass (60×) whole-genome sequencing or through family-based genotype imputation in the remaining individuals not sequenced. In an attempt to harmonize the WG-6 chip and the HT12-v3 chip we limited our replication efforts to a 397 probe subset of the 1325 whereby the probe sequences matched across the two platforms. Levels of expression were detected for 233/397 probes in 643 SAFHS individuals. Association analyses were performed using mixed-linear models as implemented in RareFAM that adjusts for a kinship matrix when performing association testing (available online from http://genome.sph.umich.edu/wiki/FamRvTest). One variant from the SAFHS replication results was classified as spurious and removed prior to testing for correlation with the initial *cis*-eQTL results as it had an effect size of >9 standard deviations of an inverse-normalized distribution of gene expression levels.

### Validation of low-frequency variants with bespoke genotyping

We selected 10 low-frequency SNPs associated with *cis*-eQTLs and 1 low-frequency lactic dehydrogenase variant for genotyping at LGC Genomics, UK. For 9/11 SNPs we obtained >99% concordance overall. There were two that were returned as monomorphic (both *cis*-eQTL variants) (Supplementary Material, Table S17).

## Supplementary Material

Supplementary Material is available at *HMG* online.

*Conflict of Interest statement:* None declared.

## Funding

This work was supported by Wellcome Trust grants: 083270/Z/07/Z and 090367/Z/09/Z. A.W. and T.F. are supported by the European Research Council grant: SZ-50371-GLUCOSEGENES-FP7-IDEAS-ERC. M.T., M.W. and A.M. are supported by the Wellcome Trust Institutional Strategic Support Award (WT097835MF). M.T. is also supported through the Wellcome Trust grant WT090367MA. M.M. is a Wellcome Trust Senior Investigator and is supported by the Wellcome Trust grant 098381. This research was supported in part by the Intramural Research Program of the NIH, National Institute on Aging (ZO1-AG000947 and Z01-AG000185) and in part by the UK Medical Research Council. The InCHIANTI study baseline (1998–2000) was supported as a ‘targeted project’ (ICS110.1/RF97.71) by the Italian Ministry of Health and in part by the US National Institute on Aging (contracts: 263 MD 9164 and 263 MD 821336). A portion of this study utilized the high-performance computational capabilities of the Biowulf Linux cluster at the National Institutes of Health, Bethesda, Md. (http://biowulf.nih.gov). We thank the T2D-GENES consortium for whole-genome sequencing in the San Antonio Family Heart Study. Sequence data were generated by the T2D-GENES consortium with support from NIH/NIDDK U01's DK085501, DK085524, DK085526, DK085545 and DK085584. Funding to pay the Open Access publication charges for this article was provided by the Wellcome Trust.

## Supplementary Material

Supplementary Data
