# Supplementary material for: Whole-genome sequencing to understand the genetic architecture of common gene expression and biomarker phenotypes
Source: Hum Mol Genet. 2014 Nov 6;24(5):1504–12. doi: 10.1093/hmg/ddu560 (PMC4321449; doi:10.1093/hmg/ddu560)
Supplement: Supplementary Data [file supp_24_5_1504__index.html]

Whole-genome sequencing to understand the genetic architecture of common gene expression and biomarker phenotypes — Whole-genome sequencing to understand the genetic architecture of common gene expression and biomarker phenotypes — Supplementary Data 

# Whole-genome sequencing to understand the genetic architecture of common gene expression and biomarker phenotypes

## Supplementary Data

Supplementary Data

**Files in this Data Supplement:**

- Supplementary Data - Doc file
- Supplementary Tables - xls file
