# Supplementary material for: Whole-genome sequencing to understand the genetic architecture of common gene expression and biomarker phenotypes
Source: Hum Mol Genet. 2014 Nov 6;24(5):1504–12. doi: 10.1093/hmg/ddu560 (PMC4321449; doi:10.1093/hmg/ddu560)

**SUPPLEMENTARY MATERIALS - FIGURES**

| **Figure S1**. The average percentage of the genome covered at different read depths across the 680 InCHIANTI subjects | **Page 2** |
| --- | --- |
| **Figure S2**. The fraction of SNPs found in HapMap, 1000 Genomes Omni 2.5 genotyping array and indels present in the 1000 Genomes project phase 1 indel datasets, binned by catalogued minor allele frequency. | **Page 3** |
| **Figure S3.** The fraction of variants discovered through low-pass sequencing that overlap with variants identified through high-pass sequencing and the fraction of variants discovered through high-pass sequencing that overlap with variants identified through low-pass sequencing. | **Page 5** |
| **Figure S4**. Plot of overall sequence-to-chip genotype concordance post Beagle refinement of the 673 “concordant” subjects. | **Page 7** |
| **Figure S5**. Sequence to Illumina HumanHap550 genotyping concordance for 673 individuals stratified by genotype as defined on chip. | **Page 8** |

**Figure S1**. The average percentage of the genome covered at different read depths across the 680 InCHIANTI subjects


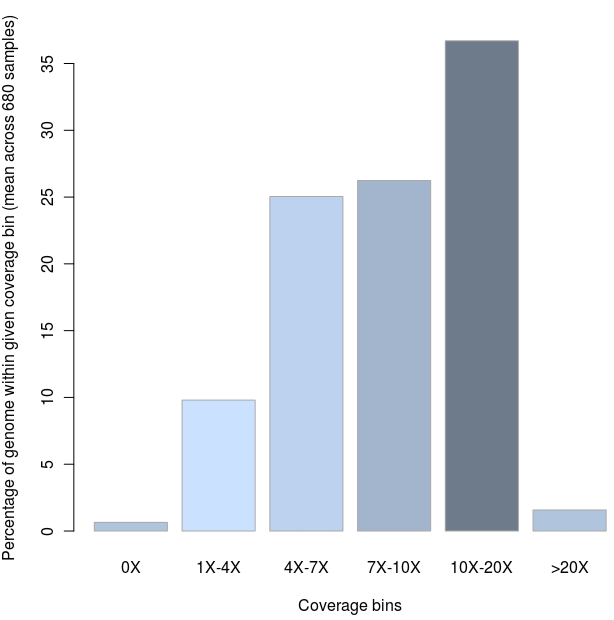


**Figure S2**. The fraction of SNPs found in HapMap, 1000 Genomes Omni 2.5 genotyping array and indels present in the 1000 Genomes project phase 1 indel datasets, binned by catalogued minor allele frequency. **a)** 1,294,681 (97.4%) SNPs present in HapMap Phase 3 release 3: 1,329,031 SNPs from CEU, GBR, TSI and Intersecting populations; **b)** 1,677,600 (76.9%) SNPs with 1,038,907 novel to dbSNP 135 present in 2,181,344 SNPs with 1,504,747 novel to dbSNP 135) on the 1000 Genomes Omni2.5 high-density genotyping array; **c)** 831,414 (57.6%) indels with 273,595 novel to dbSNP 135 present in 1,443,514 indels with 700,122 novel to dbSNP 135 from the 1000 Genomes phase 1 dataset.

**a)**


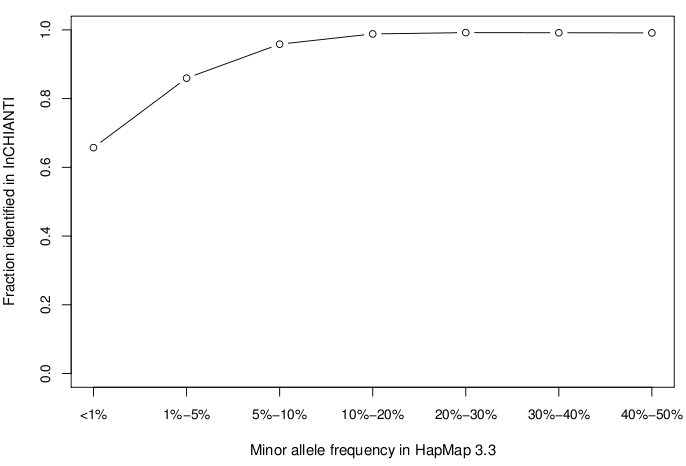


**b)**
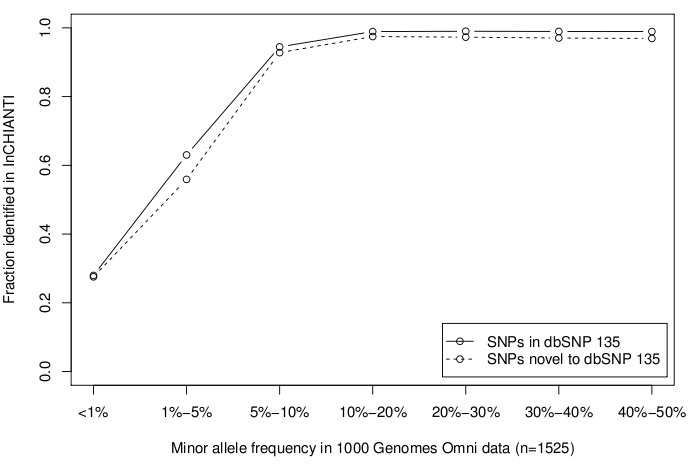


**c)**

**
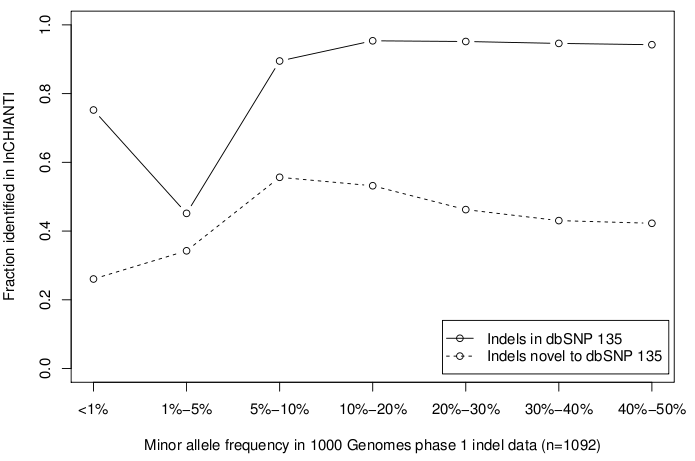
**

**Figure S3. a**) The fraction of 10,389 variants discovered through low-pass sequencing (9,402 SNPs and 987 indels) that overlap with 10,167 variants identified through high-pass sequencing (9,342 SNPs: 99.4% and 825 indels: 84%); **b)** the fraction of 11,649 variants discovered through high-pass sequencing (10,609 SNPs and 1,040 indels) that overlap with 10,167 variants identified through low-pass sequencing (9,342 SNPs: 88.1% and 825 indels: 79.3%)

a)
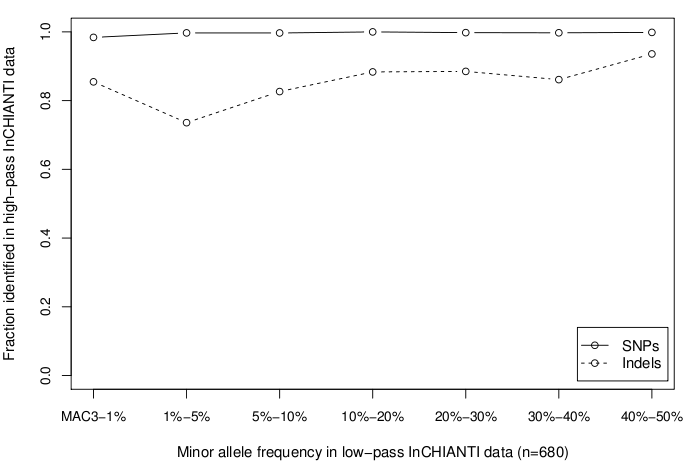


b)
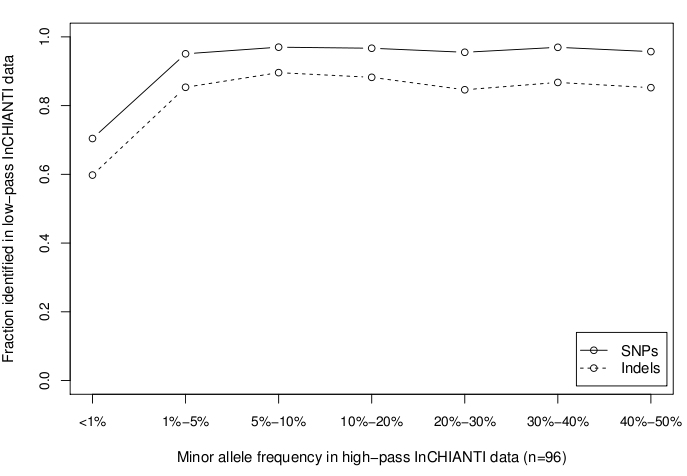


**Figure S4**. Plot of overall sequence-to-chip genotype concordance post Beagle refinement of the 673 “concordant” subjects. Subjects are ranked (indexed) in order of least to most concordance with the microarray *post* Beagle Imputation.


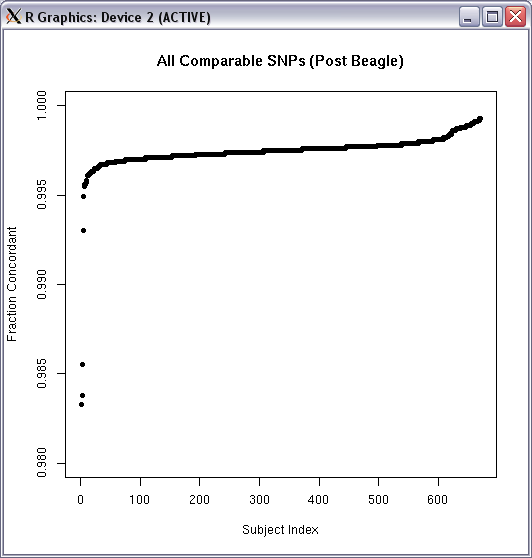


**Figure S5**. Sequence to Illumina HumanHap550 genotyping concordance for 673 individuals stratified by genotype as defined on chip. Red = common homozygous genotypes on chip; Blue = heterozygous genotypes on chip; Black = rare homozygous genotypes on chip. **a)** before beagle phasing and imputation; **b)** after beagle phasing and imputation. Subjects are ranked (index) in order of least to most concordant *prior* to beagle imputation overall.

**a)**


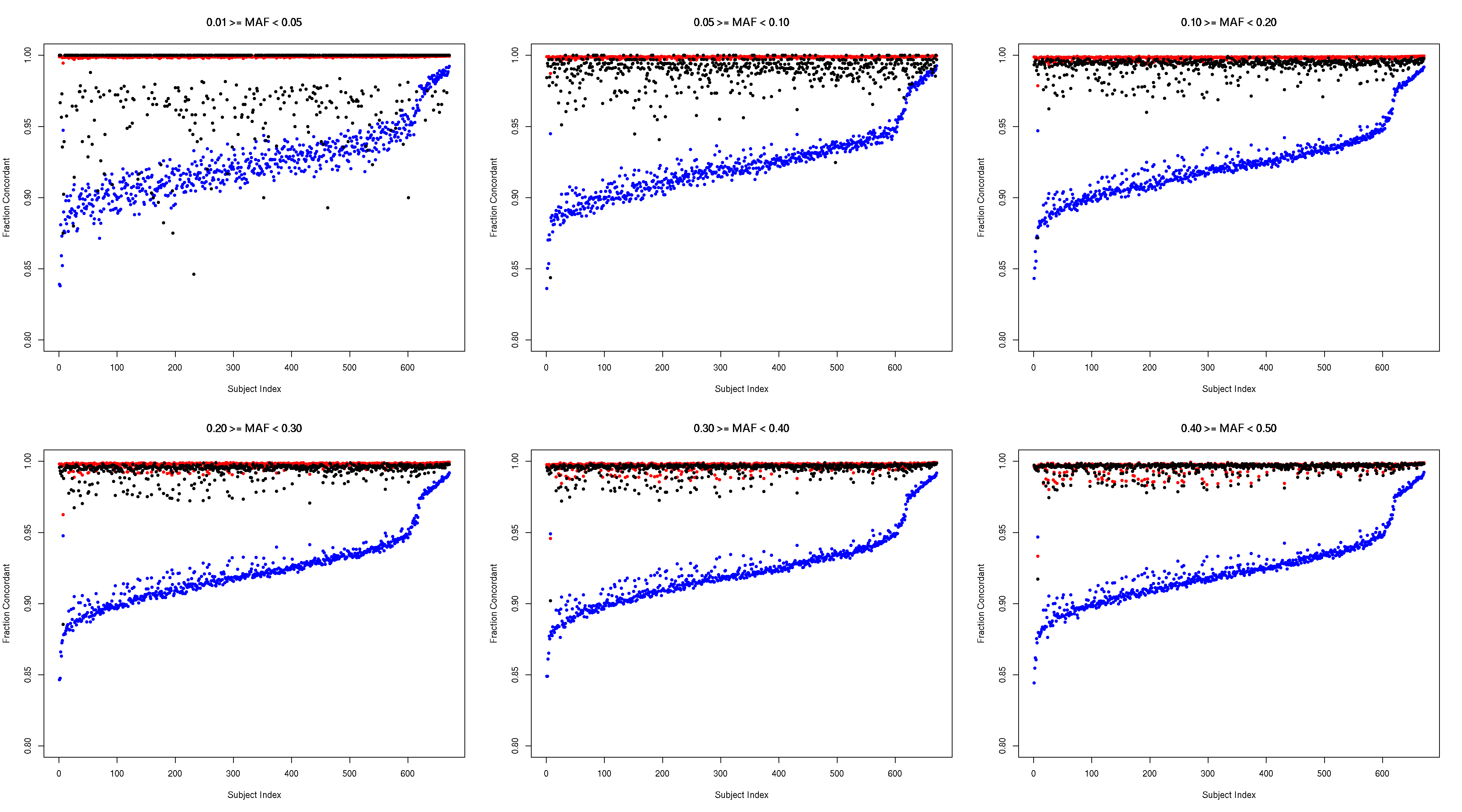


**b)**


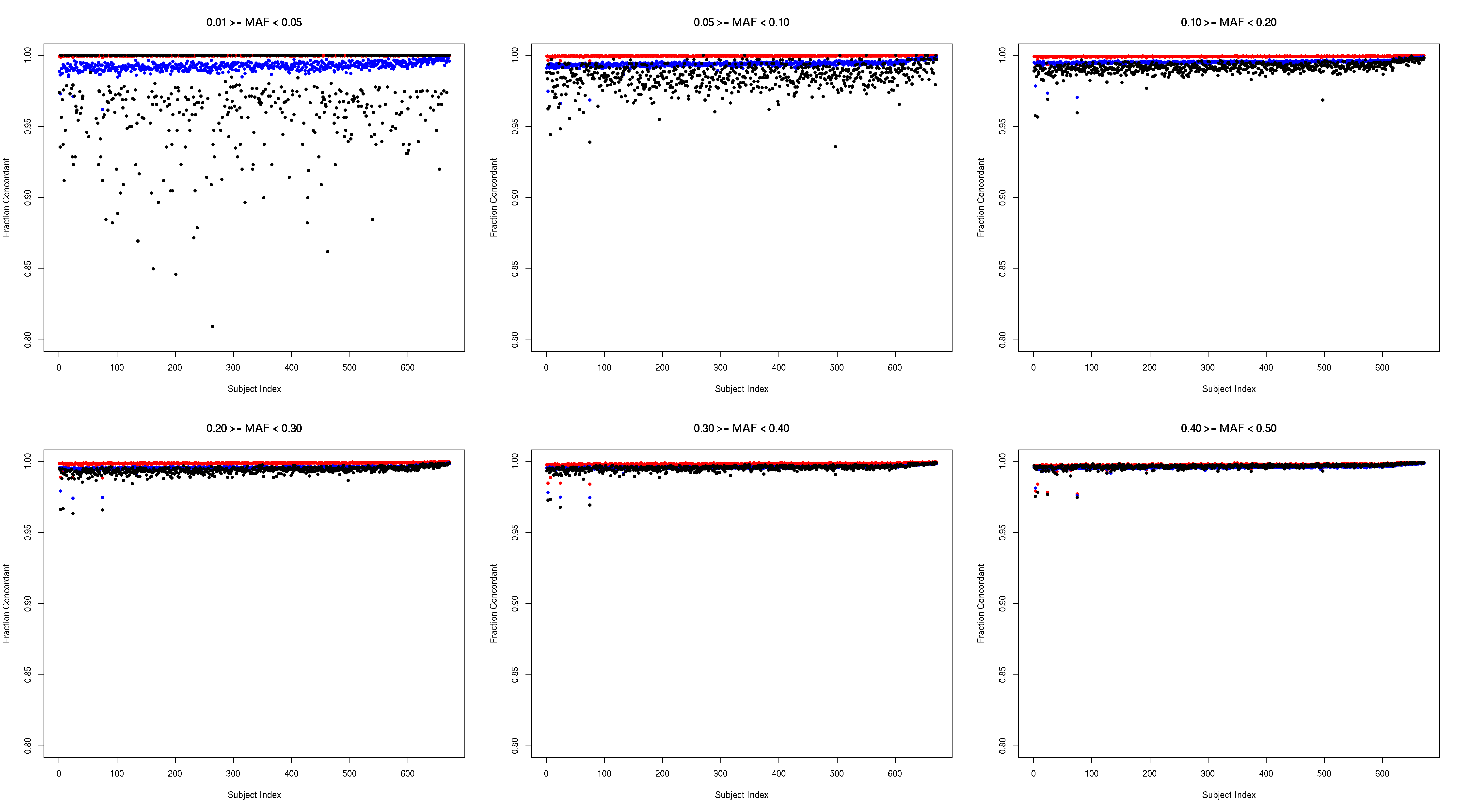

Supplement: Supplementary Data [file supp_ddu560_ddu560supp.doc]
